# Editorial: Frontiers in the Pharmacological Manipulation of Intracellular cAMP Levels

**DOI:** 10.3389/fphar.2016.00004

**Published:** 2016-01-29

**Authors:** George S. Baillie, Frank Christian, Apostolos Zarros

**Affiliations:** Gardiner Laboratory (535), Institute of Cardiovascular and Medical Sciences, University of GlasgowGlasgow, UK

**Keywords:** cyclic adenosine monophosphate, cAMP, cellular function, signaling, homeostasis, disease, pharmacological manipulation, therapeutic applications

Cyclic adenosine monophosphate (cAMP) is a second messenger of paramount biological importance, involved in the regulation of a significant number of cellular functions *via* the cAMP-dependent intracellular signal transduction pathways. Being the first second messenger described (Rall and Sutherland, 1958), cAMP has been extensively studied throughout the years and has revealed mainstream mechanisms of cellular signaling (Beavo and Brunton, 2002; Baillie et al., 2005; McCormick and Baillie, 2014). The aim of this Research Topic was to attract contributions that highlight emerging ideas in the cAMP field that: (i) describe its role in cellular function and homeostasis, (ii) present the current approaches to its pharmacological manipulation, and (iii) clarify its central role in the development of more targeted therapeutic approaches toward a spectrum of diseases.

To our belief, this aim has been successfully fulfilled. The present collection of articles highlights, in a representative (but certainly not exhaustive) way, the research activity and emerging concepts in the field, while it also reveals the therapeutic potential that targeted pharmacological manipulation of intracellular cAMP levels could exert on a number of pathological conditions. We organized the papers of this Research Topic/e-Book into three sections (A–C).

The first section (A: “*Frontiers in the role of cAMP in cellular function, homeostasis and disease”*) opens with a very informative review article on the reciprocal regulation between cAMP signaling and the ubiquitin-proteasome system, as well as its relevance to certain human diseases (Rinaldi et al.). A more focused perspective on the importance of cAMP in cardiac physiology and pathophysiology is presented by the review article of Boularan and Gales and the mini review article of Froese and Nikolaev; both articles provide an up-to-date account of recent developments in the field and should be considered by readers as complementary to each other. Later in section A, readers will discover an elegant mini review on the role of cAMP signaling in neural plasticity, learning and memory (Lee), that summarizes findings generated by research on the fruit fly *Drosophila melanogaster* and briefly highlights implications of this signaling pathway to potential therapeutic applications. Section A concludes with an original article on the role of cAMP signaling in human trophoblast fusion (Gerbaud et al.), and with a perspective article on the spatiotemporal regulation of cAMP signaling in blood platelets (Raslan et al.).

The second section (B: “*Approaches to the study and pharmacological manipulation of intracellular cAMP levels”*) includes an informative review article on state-of-the-art methodologies which can be utilized toward the study of cAMP-mediated signal transduction *via* G-protein coupled receptors (Wright et al.), as well as two excellent review articles on the pharmacological manipulation of intracellular cAMP levels through interference of the molecular interactions between the A-kinase anchoring proteins and other signaling protein intermediates (Calejo and Taskén; Nygren and Scott). This section also accommodates a contribution by Röck et al. with an original research article in which analysis of protein kinase A (PKA) dynamics following the integration of patient mutations into its catalytic (PKAc) and regulatory (RIa) subunits is presented. The section is completed with two analytical review articles that present recent developments in the utilization of genetically encoded tools for cAMP measurement in health and disease (Paramonov et al.; Patel and Gold).

The third section (C: “*Potential therapeutic applications”*) is introduced with a perspective article on the role of patients' sex toward a more targeted (cAMP pathway-regulated) therapeutic approach to brain tumors (Warrington et al.), followed by two original research articles on the role of cAMP signaling in the context of hematological malignancies and the potential therapeutic applications that could arise through its pharmacological manipulation (Dong et al.; Fernández-Araujo et al.). The current Research Topic / e-Book is concluded with a well-written review article on cAMP signaling in trypanosomatids that provides an overview of the complex functions of cAMP in these protozoan parasites, as well as a fine perspective on how potential targets for the trypanosomatid-specific cAMP pathway could provide efficient therapeutics for serious human diseases such as the sleeping sickness and other trypanosomatid-induced pathological entities (Tagoe et al.).

The recruited articles cover multiple aspects of the ongoing research in the cAMP field and allow an appreciation of the difficult task ahead in fully-understanding the complexity of this ubiquitous signaling cascade and taming it to develop efficient therapeutic applications. Hopefully, readers will consider this collection of papers as a small step toward these goals.

## Conflict of interest statement

The authors declare that the research was conducted in the absence of any commercial or financial relationships that could be construed as a potential conflict of interest.
